# Expression of Genes Encoding the Enzymes for Glycogen and Trehalose Metabolism in L3 and L4 Larvae of* Anisakis simplex*

**DOI:** 10.1155/2015/438145

**Published:** 2015-12-08

**Authors:** E. Łopieńska-Biernat, E. A. Zaobidna, M. Dmitryjuk

**Affiliations:** Department of Biochemistry, Faculty of Biology and Biotechnology, University of Warmia and Mazury, Oczapowskiego 1A, 10-917 Olsztyn, Poland

## Abstract

Trehalose and glycogen metabolism plays an important role in supporting life processes in many nematodes, including* Anisakis simplex*. Nematodes, cosmopolitan helminths parasitizing sea mammals and humans, cause a disease known as anisakiasis. The aim of this study was to investigate the expression of genes encoding the enzymes involved in the metabolism of trehalose and glycogen—trehalose-6-phosphate synthase (TPS), trehalose-6-phosphate phosphatase (TPP), glycogen synthase (GS), and glycogen phosphorylase (GP)—in stage L3 and stage L4 larvae of* A. simplex*. The expression of mRNA all four genes,* tps*,* tpp*,* gs, *and* gp*, was examined by real-time polymerase chain reaction. The* A. simplex* ribosomal gene (*18S*) was used as a reference gene. Enzymatic activity was determined. The expression of trehalose enzyme genes was higher in L3 than in L4 larvae, but an inverse relationship was noted for the expression of* gs *and* gp *genes.

## 1. Introduction

The trehalose synthesis pathway in nematodes proceeds with the participation of trehalose-6-phosphate synthase/trehalose-6-phosphate phosphatase (TPS/TPP). Biochemical and molecular data for these enzymes are available for several numbers of species. TPS data are available for the following nematodes:* Aphelenchus avenae*,* Ascaris suum*,* Brugia malayi*,* Caenorhabditis elegans*,* Ditylenchus africanus*,* Heterodera glycines*,* Heterorhabditis bacteriophora*,* Onchocerca volvulus, *and* Panagrolaimus davidi *[1–9]. TPP activity has been reported in* A. suum*,* B. malayi,* and* C. elegans* [10–12]. Studies investigating TPS and TPP have revealed that TPS activity is a limiting factor in trehalose synthesis because TPP has high affinity for trehalose-6-phosphate and hydrolyzes it very quickly to trehalose [13]. Recent studies indicate that, in* C. elegans*, trehalose increases longevity, prolongs the reproductive period, and contributes to heat tolerance. The inactivation of* tps-1* and* tps-2* genes at the cDNA level shortens life expectancy and increases sensitivity to heat stress in* C. elegans* [14], whereas mutations (knockdown) in the* gob-1* gene in the secondary pathway of trehalose synthesis cause intestinal defects in nematodes and contribute to their mortality [10]. The above studies demonstrated that death does not occur in nematodes due to an absence of trehalose and the resulting toxicity of accumulated trehalose-6-phosphate. Those observations provide valuable inputs for research into effective drugs for the treatment of parasitic diseases in humans and animals [4, 11, 15].

Similar to other organisms, glycogen synthase (GS) in nematodes plays a major role in the synthesis of glycogen, whose properties have been characterized in a limited number of nematode species:* A. suum*,* C. elegans*,* Litomosoides carinii, *and* Steinernema feltiae* [16–19]. In nematodes, glycogen levels drop during starvation and increase during feeding [20]. GS regulates the level of polysaccharides, which are essential for the survival of parasites [21]. The* Sf-gsy-1* gene has been identified in* S. feltiae*. It has been hypothesized that, during dehydration, trehalose synthesis is intensified at the expense of glycogen synthesis, which is largely controlled by the suppression of the* Sf-gsy-1* gene [19]. Glycogen phosphorylase (GP) is an essential enzyme that regulates the uptake and utilization of glycogen in nematodes [22].* Gp* genes have been found in draft genome sequences of* Trichinella spiralis* and* Loa loa* [23].

Trehalose can be synthesized from glucose, but the interactions between glycogen and trehalose have not been explored to date. There is no evidence that nematodes use glycogen in trehalose synthesis. However, glucose-1-phosphate, a product of GP, can be converted to glucose-6-phosphate or uridine diphosphoglucose, which are substrates for TPS [13]. Both pathways should be investigated, and the key point for parasite control should be determined to indicate which pathway plays a more significant role for the parasite.

Carbohydrate metabolism plays a vital role in sustaining life processes in many nematodes, including* A. simplex*. The properties of TPS and TPP have been described in stage L3 larvae of* A. simplex* [24]. The activity of GPa and b has been studied in* A. simplex* [25], but mRNA levels have not been investigated to date. The synthesis of trehalose and glycogen protects developing parasites against external stressors and enables them to survive in harsh conditions [19, 26]. The growing incidence of anisakiasis caused by the consumption of raw fish has prompted research into new antiparasitic drugs. The blockade of genes encoding trehalose synthesis enzymes contributes to nematode death, and this observation can be used to develop new antiparasitic treatments [2, 10, 11]. Environmental stressors also reduce the expression of the GS gene, which leads to an increased synthesis of trehalose [19]. The above demonstrates that the synthesis of both saccharides can be controlled at the molecular level in nematodes. The aim of this study was to investigate and compare the expression of genes encoding the enzymes involved in trehalose synthesis (TPS and TPP), glycogen synthesis (GS), and breakdown (GP) in L3 and L4 larvae of* A. simplex*. This study also tests the hypothesis formulated in our previous work [25], namely, that changes in host environments accompanied by metabolic adaptations associated with changes in enzyme activities probably also induce changes at the genetic level.

## 2. Materials and Methods

### 2.1. Parasite Material

L3 larvae of* A. simplex* were collected from fresh Baltic herring (*Clupea harengus*). The larvae were axenized in antibiotic solution [27] and rinsed several times in sterile saline solution (0.65%). L3 larvae were the starting material for the* in vitro* cultures of L4 larvae of* A. simplex*, developed according to the method proposed by Iglesias et al. [28]. The larvae were placed in sterile containers, frozen in liquid nitrogen, and stored at –75°C. Samples, for enzymatic activity, were then homogenized in an electric homogenizer at a 1 : 1 (w/v) ratio in TBS buffer. The homogenate was centrifuged at 1500 g for 15 min at 4°C. The supernatant was used to assay for enzymes (TPS, TPP).

### 2.2. Total RNA Isolation and Reverse Transcription (RT)

Total RNA was extracted from both larval stages with the Total RNA Kit (A&A Biotechnology) according to the manufacturer's protocol. The quantity and purity of isolated RNA were determined by spectrometry using the Nano Drop 1000 spectrophotometer (Thermo Scientific) and by 1.5% formaldehyde-agarose gel electrophoresis. RT was performed using the reverse transcriptase TranScriba Kit (A&A Biotechnology, Poland). To eliminate the contamination of genomic DNA, RNA extracts were treated with DNAse/RNAse-free eraser (A&A Biotechnology), and the mixture was incubated at 37°C for 15 min. Total RNA (2 *µ*g) was reverse-transcribed to cDNA using specific reverse primers (Table 1). The RT reaction was carried out at 37°C for 45 min and at 72°C for 10 min. The cDNA product with a final volume of 20 *µ*L was stored at –20°C until further analysis. The primers used in the experiment were designed with Primer3 (http://frodo.wi.mit.edu) based on gene sequences in the GenBank database (http://www.ncbi.nlm.nih.gov/). The* A. simplex* ribosomal gene (*18S*) (U81575) was used as a reference gene.

### 2.3. Sequencing and Cloning cDNA of* tps1*,* tpp*,* gs*, and* gp* from L3 Larvae of* A. simplex*

Polymerase chain reaction (PCR) was performed with the use of primer sequences shown in Table 1. The reaction mixture consisted of 5 *µ*L of cDNA, 25 *µ*L of Master Mix Plus High GC (A&A Biotechnology), 0.1 *µ*L of the corresponding forward and reverse primers (Table 1) with the concentration of 100 mM, and 23.8 *µ*L of nuclease-free water. PCR was performed in a gradient thermocycler (Applied Biosystems) with the following temperature-time profile: 94°C, 3 min; (94°C, 30 s; 50°C (58, 55, 53°C), 1 min; 72°C, 30 s) ×40 cycles; 72°C, 7 min. PCR products were subjected to electrophoresis on 2% agarose gel containing 0.01% ethidium bromide. During cloning into the pJET1 vector (3.1 kb; Fermentas, Burlington, Ontario, Canada), a nonpurified DNA fragment was reacted with 0.5 *µ*L of Walk DNA Polymerase (thermostable polymerase Pwo; A&A Biotechnology) for 5 min at 70°C in a Gradient thermocycler (Eppendorf). After electrophoresis, the products were isolated from the 2% gel and purified using the Gel-Out Kit (A&A Biotechnology). PCR products were thus obtained with blunt ends suitable for cloning into the vector. The ligation reaction was adjusted to 20 *µ*L. The ligation mixture consisted of 1 *µ*L of pJET1 DNA, 9 *µ*L of the corresponding* tps1*,* tpp*,* gs,* and* gp* gene fragments, 1 *µ*L of DNA T4 ligase (A&A Biotechnology), and 9 *µ*L of water. Ligation was performed for 18 min at room temperature. The ligation mixture was transformed into competent cells of* Escherichia coli* TOP10F′ (Novagen, Madison, WI, USA). Competent cells were prepared using the* E. coli* Transformer Kit (A&A Biotechnology). In the transformation process, 100 *µ*L of competent cells was used, to which the entire volume of the ligation mixture was added. The sample was placed on ice for 60 min and electroporated in Sonic3 (Polsonic, Poland) for 3 s. The prepared samples were distributed on plates with LA (Luria Agar) solid medium with ampicillin (50 *µ*L/mL). The plates were incubated for 16 h at 37°C. The potential presence of clones in the colonies was checked for* gs, gp *and* tps1, tpp*  gene fragments by PCR using primers and reaction conditions. Transformant colonies where the presence of the gene had been confirmed were transferred to 5 mL of liquid LB (Luria Broth) medium with ampicillin (50 *µ*L/mL) and incubated for 17 h at 37°C. Recombinant plasmids were isolated from cultures using the Plasmid Mini AX Kit (A&A Biotechnology). The presence of gene fragments in the resulting plasmids was investigated by PCR based on the above protocol. PCR product sequencing was performed in the 3730xI DNA Analyzer (Applied Biosystems, Carlsbad, CA, USA).

### 2.4. Sequence Analysis of Partial* tps1*,* tpp*,* gs* and* gp* mRNA in L3 Larvae of* A. simplex*

Analyses of* tps1, tpp, gs*, and* gp* and sequence alignment were performed in Geneious 7.0 created by Biomatters (available from http://www.geneious.com/).

### 2.5. Relative Quantification of Gene Expression

Relative quantification of gene expression by real-time PCR was performed to compare* tps1, tpp, gs*, and* gp* transcription levels in L3 and L4 larvae of* A. simplex*. Fold changes in target genes, normalized to* 18S* and relative to expression levels in the larvae, were calculated using the comparative Ct (2^−ΔΔCt^) method [29]. Quantitative real-time PCR was performed using SYBRGreen Taq PCR-MIX (A&A Biotechnology, Poland) according to the manufacturer's protocol. A quantity of 20 *µ*L of the reaction solution contained 1 *µ*L of the template (1 : 10 dilution of cDNA product), 10 *µ*L of SYBRGreen Taq PCR-MIX (2x), 1 *µ*L of 10 *µ*M of each primer (Table 2), 6.6 *µ*L of water, and 0.4 *µ*L of ROX Reference dye II. The following thermal cycling conditions were applied: 10 min at 95°C followed by 38 cycles of 20 s at 95°C, 20 s at 55°C, and 30 s at 72°C. The data were analyzed and normalized relative to transcript levels of 18S ribosomal RNA genes in the AB analytical application (7500v2.0). All samples were tested in triplicate in the FAST7500 LightCycler (Applied Biosystems, USA). Melting curves were developed after amplification.

### 2.6. TPS and TPP Activity

TPS activity was determined based on the method described by Giaever et al. [30] and TPP activity was marked on the method proposed by Kaasen et al. [31]. Trehalose, the end-product of these reactions, was determined by high-performance liquid chromatography according to the procedure described by Dmitryjuk et al. [7]. The activity of TPS and TPP was expressed in units (U) per mg of protein measured spectrophotometrically at 280 nm in the Nano Drop ND-1000 spectrophotometer (Thermo Fisher Scientific, Waltham, MA, USA). One unit defines the quantity of synthesized trehalose (*μ*mol) during 1 min at 37°C. The results are presented as mean values from five independent measurements with five replicates per sample.

### 2.7. Statistical Analysis

Data are expressed as mean values and standard deviations. The statistical significance of differences between samples at both developmental stages was analyzed by one-way analysis of variance (ANOVA) and Tukey's test in the SPSS 16.0 application at a significance level of *P* < 0.05. Pearson's correlation test was used for determination of the correlation between the enzymes activities of TPS, TPP, GS, and GP and the expression of* tps1*,* tpp*,* gs,* and* gp* mRNA in L4 larvae of* A. simplex*.

## 3. Results

### 3.1. Sequencing of Trehalose-6-Phosphate Synthase and Phosphatase Genes

**Figure 1:**
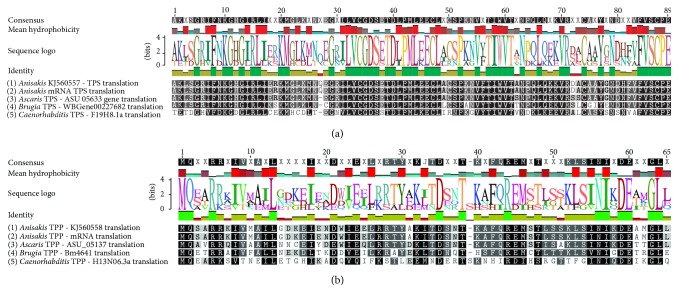
Multiple alignment of predicted partial deduced amino acid sequences of trehalose-6-phosphate synthase (a) and trehalose-6-phosphate phosphatase (b) from nematodes: third-stage (L3) larvae of* Anisakis simplex*,* Caenorhabditis elegans*,* Brugia malayi*, and* Ascaris suum*. The first line shows the consensus sequence of numbers amino acid position. Below (mean hydrophobicity) is a histogram of the hydrophobicity of individual amino acids. The line sequence logo is a consensus sequence where the size of the symbol of the amino acid determines its stability in its use position (this value also describes a line identity), and each amino acid is indicated by another color. Tinting the background of individual items in sequence on alignment determines similarity in position on all sequences.

Gene fragments encoding parts of TPS and TPP in L3 larvae of* A. simplex* were isolated and sequenced. The fragments encoding sequences for* tps* and* tpp* had a length of 268 bp and 192 bp, respectively (Figures 1(a) and 1(b)). These sequences of mRNA have not been deposited in GenBank, being too short.

### 3.2. Sequencing of Glycogen Synthase and Phosphorylase Genes

**Figure 2:**
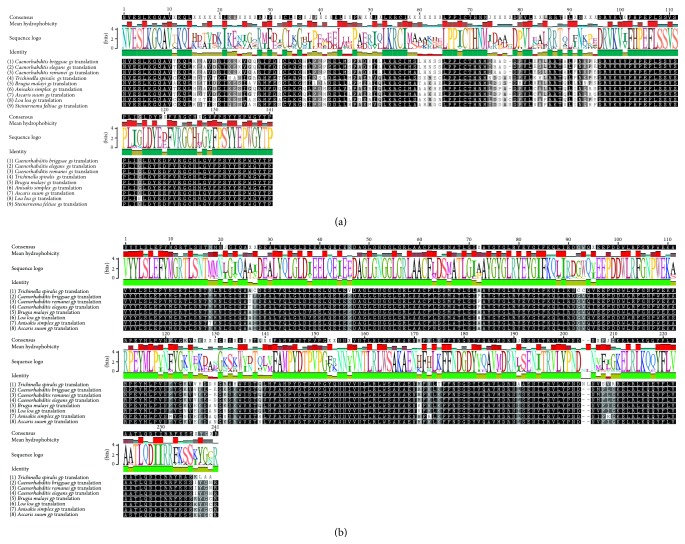
Multiple alignment of predicted partial deduced amino acid sequences of glycogen synthase (a) and glycogen phosphorylase (b) from nematodes: third-stage (L3) larvae of* Anisakis simplex*,* Loa loa*,* Caenorhabditis elegans*,* C. remanei*,* C. briggsae*,* Trichinella spiralis*,* Brugia malayi*,* Ascaris suum*, and* Steinernema feltiae*. The legend is the same as in Figure 1.

The genes encoding GS and GP in L3 larvae of* A. simplex *were isolated in part and sequenced. The fragments encoding sequences for* gs* and* gp* had a length of 485 bp and 927 bp, respectively. The analyzed fragments were deposited in GenBank (accessions numbers JX173686 and JX941465.1). Predicted translated products were 162 amino acids for AFP95337.1 and 309 amino acids for AFX58973.1 (Figures 2(a) and 2(b)).

### 3.3. Analysis of* A. simplex* Gene Expression by Real-Time PCR

**Figure 3:**
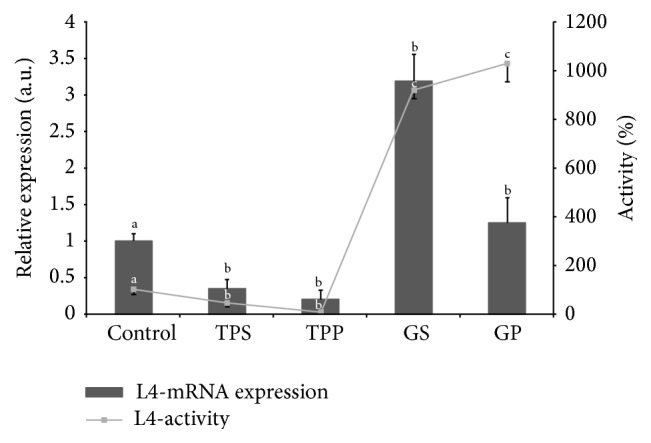
Comparison of relative activity of enzymes and expression of four genes encoding the enzymes trehalose-6-phosphate synthase (TPS), trehalose-6-phosphate phosphatase (TPP), glycogen synthase (GS), and glycogen phosphorylase (GP) using the normalization reference gene* ryb* between L3 and L4 larval stages of* Anisakis simplex*. Different letters (a, b, and c) above the bars represent significant differences between L3 (control) and L4 of* A. simplex* for gene expression and enzyme activity. ¥ Data from Łopieńska-Biernat et al. [25], *r* = 0.95 for enzymes and mRNA of trehalose metabolism, *r* = −0.8 for glycogen metabolism in L4 of* A. simplex* (*P* < 0.005) (as control 100% activity of enzymes was 0.43 ± 0.02 U/mg for TPS and 0.34 ± 0.03 for TPP U/mg in L3).

Relative quantification of the expression of four genes in the L3 and L4 larval stages was performed by real-time RT-PCR using cDNA from the total RNA of L3 and L4 larvae of* A. simplex*. The analysis confirmed differences in gene expression between L3 and L4 larvae that had been observed using semiquantitative PCR. The expression patterns were grouped into two profiles: (1) the* tps1* gene was characterized by a 3-fold increase and the* tpp* gene by a 5-fold increase in expression in L3 larvae relative to L4 larvae, and the differences between developmental stages were significant; (2) the expression of the* gs* gene was 3-fold higher and the expression of the* gp* gene was 1.2-fold higher in L4 than in L3 stages (Figure 3).

### 3.4. Activity of* A. simplex* Trehalose Synthesis Enzymes

The activity of both trehalose synthesis enzymes was higher in L3 than in L4 larvae. The activity of TPS increased 2-fold, but the activity of TPP was higher by 10-fold in L3 than in older larvae. The differences in the activity levels of both enzymes in L3 and L4 larvae were statistically significant (*P* < 0.05) (Figure 3).

The correlation between the activity of enzymes for trehalose and glycogen metabolism and their mRNA expression is shown in Figure 3. The correlation coefficient was higher (*r* = 0.9, *P* < 0.005) between trehalose synthesis enzymes and mRNA expression. Meanwhile, a moderate correlation was also obtained between enzymes of glycogen metabolism and mRNA expression values (*r* = −0.8, *P* < 0.005) (Figure 3).

## 4. Discussion

The* A. simplex* genome has been partially sequenced. Scaffolds of genomic DNA have been submitted to GenBank (http://www.ncbi.nlm.nih.gov/), but they do not reveal active genes. An analysis of expressed sequence tags in L3 larvae of* A. simplex* constitutes an important part of research. EST revealed that 20% of matched clones were highly homologous with the genes or proteins of* C. elegans* [32]. The above achievement and the completely sequenced* C. elegans* [33] facilitated and contributed to the identification of genes in* A. simplex*. Molecular research based on known homologous sequences in* C. elegans* did not produce positive results, and partial success was achieved only when degraded primers, designed based on* A. suum* and* B. malayi* sequences, were used.

Trehalose is a disaccharide that protects various organisms against environmental stressors such as dehydration, anoxia, or changes in temperature [14]. L3 larvae, which have as their natural host heterothermic organisms such as fish and cephalopods, are extremely viable and resistant. However, the fourth larval stage (L4) develops in the alimentary tract of the definitive host, that is, marine mammals, or in humans [34–36]. In recent studies,* C. elegans* was used to explore trehalose metabolism [2, 14]. Those studies investigated the expression and function of the gene encoding the TPS enzyme, which participates in trehalose synthesis in* C. elegans* and other nematode species. Two TPS encoding genes were identified in* C. elegans*:* tps-1* (4351 bp) and* tps-2* (3862 bp), and the products of both genes were classified into the glycosyltransferase 20 family [2, 37]. Two* tps* genes with very high resemblance to the* tps-2* gene of* C. elegans* were also identified in* A. avenae*, but the expression of a gene similar to* Ce-tps-1* has not yet been confirmed. Aav-TPS-2 and Ce-TPS-2 proteins exhibit 62% similarity, whereas Aav-TPS-1 and Ce-TPS-1 are characterized by 49% similarity, and the sequences of both genes in* A. avenae* and* C. elegans* show 51% similarity. The two genes encoding TPS are often not expressed in multicellular animals [9]. In this study, the* tps-1* gene in the L3 larvae of* A. simplex* was partially identified and its expression was demonstrated at the cDNA level, but the presence of the* tps-2* gene was not confirmed. The presence of a one gene encoding the TPS enzyme and only partial cDNA may be difficult to identify in* A. simplex* larvae because not all genes are active in the analyzed developmental stage and mRNA is very unstable. Our bioinformatics analysis of genomic DNA confirmed the presence of a single form of the gene, TPS1 (GenBank accession number KJ560557). The expression of the* tps* gene has not been explored to date. The partial* tps *gene in L3 larvae of* A. simplex* has a length of 268 bp. The resulting gene of* A. simplex* invasive larvae is less similar to* tps* genes in other parasites, including* O. volvulus* (421 bp) and* B. malayi* (834 bp) [2]. The translated sequenced gene in L3 larvae of* A. simplex* shows 58.5% similarity to the* Ce-tps-1* gene (Figure 1(a)). The second stage of trehalose synthesis catalyzes the TPP enzyme. The enzyme has been studied at the genetic level in two organisms:* C. elegans* and* B. malayi* where one gene (*gob-1, tpp*) encoding the TPP enzyme was identified [10, 11]. Expression of the partial* tpp* gene in L3 larvae of* A. simplex* was determined in this study, and sequence homology with genomic DNA was confirmed in 98% (GenBank accession number KJ560557). The similarity between the sequence of the translated* tpp* gene in* A. suum *and other* tpp* genes, which were used to design the primers, was only 37.4%, and the similarity between the analyzed* tpp* gene and the* gob-1* gene was only 21.1% [4, 38].

Glycogen occurs commonly in living organisms as a major source of energy [39]. In invertebrates, glycogen is a source of energy during intensified metabolic processes [40]. The glycogen synthesis pathway is highly conserved in eukaryotes, and it involves the same enzymes in different organisms, from yeasts to humans [41]. The expression of GS genes has also been studied in the limited organisms:* C. elegans*, where the activity of* gsy-1* was observed [42],* S. feltiae* (*Sf-gsy-1*) [19], and* B. malayi* [43]. The analyses conducted in this study with the involvement of specific primers supported the identification of* gs* expression at the cDNA level in L3 larvae of* A. simplex. *The partially sequenced* gs* gene in* A. simplex* has a length of 485 bp and is shorter than* gsy-1* in* C. elegans* (2427 bp) and* B. malayi* (512 bp), but longer than in* S. feltiae* (327 bp) and* Loa loa* (327 bp). The gene identified in* A. simplex* exhibits high similarity to other GS genes in different species, including 72% similarity to the* gsy-1 *gene in* C. elegans* and 73% similarity in* B. malayi* (Figure 2(a)). Changes in the expression of the* Sf-gsy-1* gene were noted in dehydrated and rehydrated* S. feltiae* parasites, which could indicate that glycogen synthesis protects nematodes against dehydration. Increased trehalose synthesis in response to desiccation stress takes place at the expense of glycogen synthesis, which is largely controlled by the suppression of the* Sf-gsy-1* gene [19]. It can be assumed that similar changes occur in L3 larvae of* A. simplex* in response to environmental stress. The analyses performed in this study with the use of specific primers supported the determination of* gp* expression at the cDNA level in L3 larvae of* A. simplex*. The partial sequence of the* gp* gene in* A. simplex* has a length of 927 bp and is shorter than the* gp* gene in* C. elegans* (2000 bp),* L. loa *(LOAG0616) (2565 bp), and* Trichinella spiralis* (2844 bp) [22]. The cluster analysis of nematodes revealed that* gp* and* gs* products in* A. simplex* exhibit high sequence similarity to analogous mRNA in parasitic nematodes (*L. loa* and* B. malayi*) and free-living nematodes (*C. elegans*) (Figure 2(b)).

The expression of trehalose and glycogen metabolism genes is difficult to discuss due to the absence of published data concerning parasitic nematodes. The activity of trehalose synthesis enzymes is correlated with the expression of genes encoding those enzymes. The activity of trehalose enzymes and their genes was higher in L3 than in L4 larvae of* A. simplex*. The demonstrated high positive correlation also confirms the crucial role of trehalose metabolism in L3 larvae of* A. simplex* (Figure 3). Sex-based differences in the expression of trehalose and glycogen genes were also observed in* C. elegans*. Male nematodes, which are smaller and more sensitive to stress, exhibit a higher expression of trehalose genes than hermaphrodites, such as young larvae of* A. simplex* [44].

The expression levels of genes responsible for glycogen breakdown and synthesis transcripts were higher in older larvae (Figure 3). An inverse relationship was noted between the predominance of GS transcripts over GP transcripts in* A. simplex* and the predominance of GP over GS in dauer larvae of* C. elegans* [42]. Similar results were reported in a previous study investigating the activity of glycogen enzymes in L4 larval stage [25], which revealed higher activity levels of GP than GS in older larvae of* A. simplex* (Figure 3).

Our results suggest that L3 larvae produce trehalose rather than glycogen, whereas the opposite could be true in L4 larvae of* A*.* simplex*. The above hypothesis was formulated in view of the higher expression of trehalose synthesis genes and lower expression of glycogen synthesis genes in L3 larvae compared to in L4 larvae of* A. simplex* (Figure 3). Our results and fungus* Magnaporthe oryzae *[45] suggest that probably glycogen breakdown (*gp)* is a significant factor in regulation genes expression as source glucose and substrate for synthesis trehalose or synthesis trehalose (*tpp*) as toxicity of accumulated T6P [10, 15]. The parasite can probably be controlled in the metabolic pathways of both sugars, trehalose and glycogen.

Trehalose and glycogen metabolism plays a vital role in most living organisms [14, 39]. The expression of* tps1*,* tpp,* and* gs* and* gp* genes in* A. simplex *points to the importance of trehalose and glycogen metabolism in the life processes of parasites and expands our knowledge of nematode genomes.

## Figures and Tables

**Table 1:** Nucleotide sequences of primers for PCR amplification of genes encoding the enzymes for glycogen metabolism.

Primers	Names	Primers (5′-3′)	Annealing temperature (°C)	Size of PCR product (bp)
For.	*tps1s*	GGGTCTTGGGAGATCAACA	50	268
Rev.	*tps1as*	TGCTGCTTTGGTGTCAACTC		

For.	*tpps*	AGCGTTATTCAGTGGCTCGT	58	192
Rev.	*tppas*	CATGGCACTCTTTGTTGGTG		

For.	*gsFor*	CAAYGTBGARTCVCTSAAAGGHC	55	485
Rev.	*gsRev*	ATRAARCADCCRAADCCDGAVAG		

For.	*gps*	GATCGCCGTAAGCAGATCAGCGT	53	927
Rev.	*gpas*	CGGCATCCATACTTGCTTGATTTG		

**Table 2:** Primer sequences used for semiquantitative and real-time PCR.

Gene	Accession number	Primers (5′-3′)	Size of PCR product (bp)
*tps*	^*∗*^/KJ560557	For. GAAGTTACGTCAATCAATATGAGAAGG/ACTACGCATCACAAGCAACG	163
		Rev. TTCAGGTCCACCCACCCATC/CGCTTGACGTATCAATGGAA	

*tpp*	^*∗*^/KJ560558	For. GTTTTCGCTTGTCTGCTCACG/TACGAAAGCATTCCAACGTG	193
		Rev. TCGTTAGCGGCATTTCCTG/ATCGGATGATACGCTGCAAG	

*gs*	JX173686	For. GCCACCGATTTGCACACACAA	173
		Rev. CAGCCACGCAAATTCCTCA	

*gp*	JX941465	For. GGATTCGCACACAACAATACTA	193
		Rev. RCTGCGTCTTCTCGATCTCTTG	

*18S*	U81575	For. ACCAGTAACGAAAGCGTGTG	211
		Rev. GCTGTTGGAAGGAAGAACGA	

^*∗*^This sequence data was used to design primers for real-time PCR.
